# A Case Report of Marfan Syndrome Presenting With Atypical Chest Pain: A 28-Year-Old Male With Non-ST-Elevation Myocardial Infarction (NSTEMI)

**DOI:** 10.7759/cureus.22040

**Published:** 2022-02-08

**Authors:** Tayyab Cheema, Malek Balek, Patrick Smith, Saad Hanan

**Affiliations:** 1 Internal Medicine, West Suburban Medical Center, Chicago, USA; 2 Cardiology, West Suburban Medical Center, Chicago, USA; 3 Department of Medicine, Saint James School of Medicine, Park Ridge, USA

**Keywords:** marfan syndrome, multi-disciplinary teams, aortic pseudoaneurysm, non-st segment elevation myocardial infarction (nstemi), fibrillin-1, marfan disease

## Abstract

Marfan syndrome is a rare autosomal dominant disorder of the connective tissue. It results in a mutation in the Fibrillin-1 protein gene. We present a case of  Marfan’s syndrome in a young adult with life-threatening, sudden onset of chest pain secondary to a non-ST elevation myocardial infarction (NSTEMI) in the setting of an aortic pseudoaneurysm. Taking into consideration potential life-threatening underlying processes, a thorough and detailed methodology must be undertaken when encountering chest pain in a Marfan’s syndrome patient. This case highlights the importance of utilizing a multi-disciplinary approach to the complexities of Marfan syndrome.

## Introduction

Marfan syndrome is a rare autosomal dominant connective tissue disease occurring in approximately one out of 5000 individuals in the global population resulting from a mutation in the Fibrillin-1 protein gene (*FBN1*) [1,2]. Abnormal Fibrillin-1, an extracellular matrix (ECM) protein and essential component of microfibrils within the ECM, cause ECM structural changes and dysregulation of matrix homeostasis, resulting in the wide variety of clinical manifestations of Marfan syndrome, including issues in the eyes, joints, and most lethally, within blood vessels. Although the most identifiable signs on the exam are often subluxation of the lenses and musculoskeletal abnormalities, including arachnodactyly, the patient’s long-term prognosis is determined by cardiovascular manifestations [3]. 

Given Marfan syndrome results in abnormal microfibril structure and ECM changes, and blood vessels are primarily connective tissues, it is no surprise that individuals with Marfan syndrome have a disproportionate frequency of aortic disease. With the high pressures produced by the left ventricle and experienced in the aorta, the aortic root must resist deformation under substantial forces. Wild-type Fibrillin-1 helps provide structural support to the aortic walls as an element of microfibrils, and increasing evidence supports its role in ECM homeostasis preventing harmful remodeling [1]. In Marfan syndrome, the aorta is rendered less distensible, resulting in structural failure under high stresses rather than elastic distension and appropriate return to its original shape [3,4]. Thus, individuals with Marfan syndrome would be expected to develop structural issues from lower forces within the aorta than those with a normal genotype and undergo remodeling predisposing the tissues to structural failure at an increased rate compared to individuals without dysfunctional Fibrillin-1. As a result of their increased rate of cardiovascular disease development, individuals with Marfan syndrome require close monitoring to screen for cardiovascular complications and frequently require surgical intervention. 

We present a case of Marfan syndrome with a history of Type B dissection previously treated with total aortic root and arch replacement and valve replacement, who successfully underwent repair of an aortic arch pseudoaneurysm and concomitant evacuation of a thrombus. An aggressive surgical strategy followed by life-long cardiovascular monitoring is warranted to prolong the survival of such patients.

## Case presentation

The patient is a 28-year-old male with a past medical history of Marfan syndrome, status post aortic root and total arch replacement and descending aortic dissection repair in 2018 with mechanical aortic valve replacement due to regurgitation, who experienced sudden onset of chest pain during a video conference call. He had been sitting calmly working on his computer when the pain began. His pain was substernal and without radiation, described as tightness and heaviness lasting for 15 minutes without any other associated symptoms. The patient denied experiencing any strong emotions during the video call. He denied any relief with continued rest or any exacerbating factors. He reported no recent medication changes and denied any recent recreational drug use. Upon presentation to our medical center, the patient’s heart rate was 90 beats per minute, blood pressure (BP) of 118/77 mmHg, respiratory rate (RR) of 20/min, and he was afebrile. His physical exam was remarkable for scarring from right lens repair due to lens dislocation, pectus excavatum with a midline sternotomy scar with extensive keloid formation, a systolic ejection murmur with click auscultated most prominently at the right upper sternal border, and long and thin phalanges. His laboratory findings were generally unremarkable, except for a markedly elevated initial troponin of 8.87. The urine drug screen was negative. ECGs were negative for any evidence of ischemia or infarction. His transthoracic echocardiogram visualized the mechanical prosthesis in the aortic position and appeared to be functioning normally, demonstrated mild concentric left ventricular hypertrophy, and left ventricular ejection fraction was estimated to be 45-50%. 

**Figure 1:**
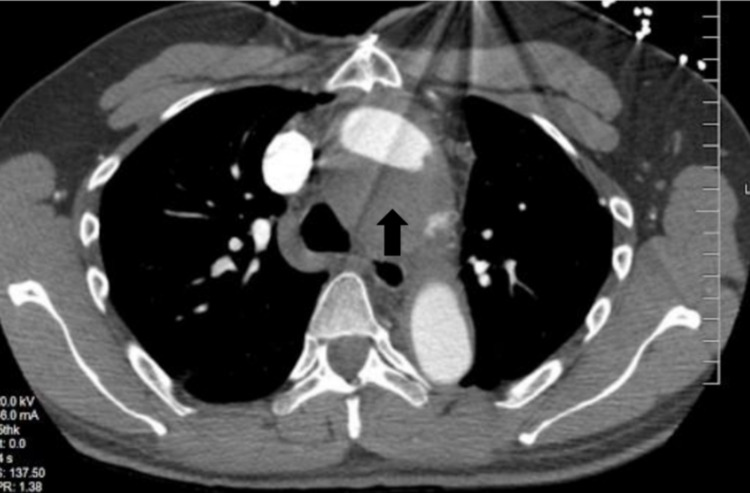
CT angiogram of the chest (axial view) Note the pseudoaneurysm inferior to true aortic lumen with the arrow pointing toward the thrombosed component.

**Figure 2:**
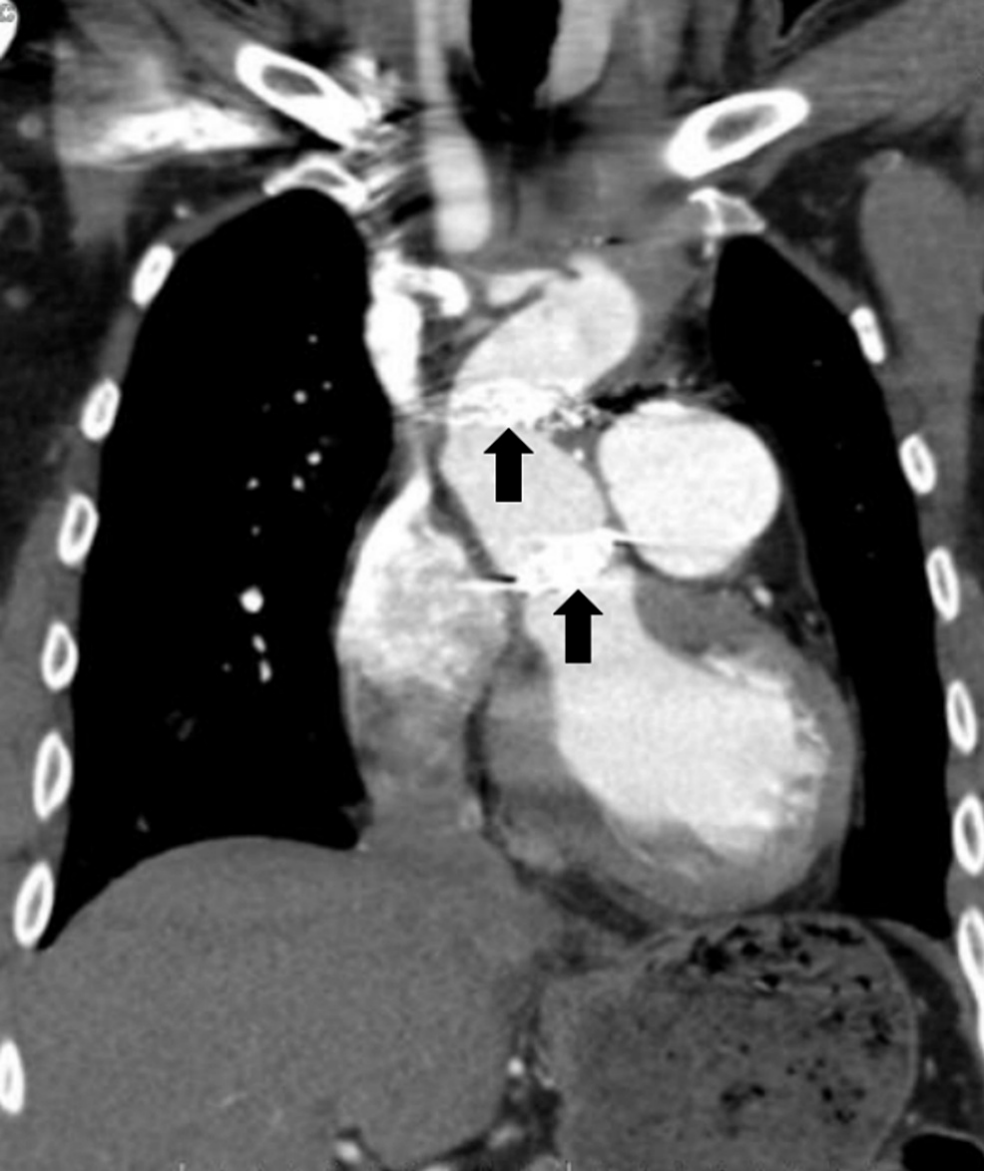
CT angiogram of the chest (coronal view) The prior aortic repair anastomotic sites can be easily visualized in this plane (indicated by arrows).

**Figure 3:**
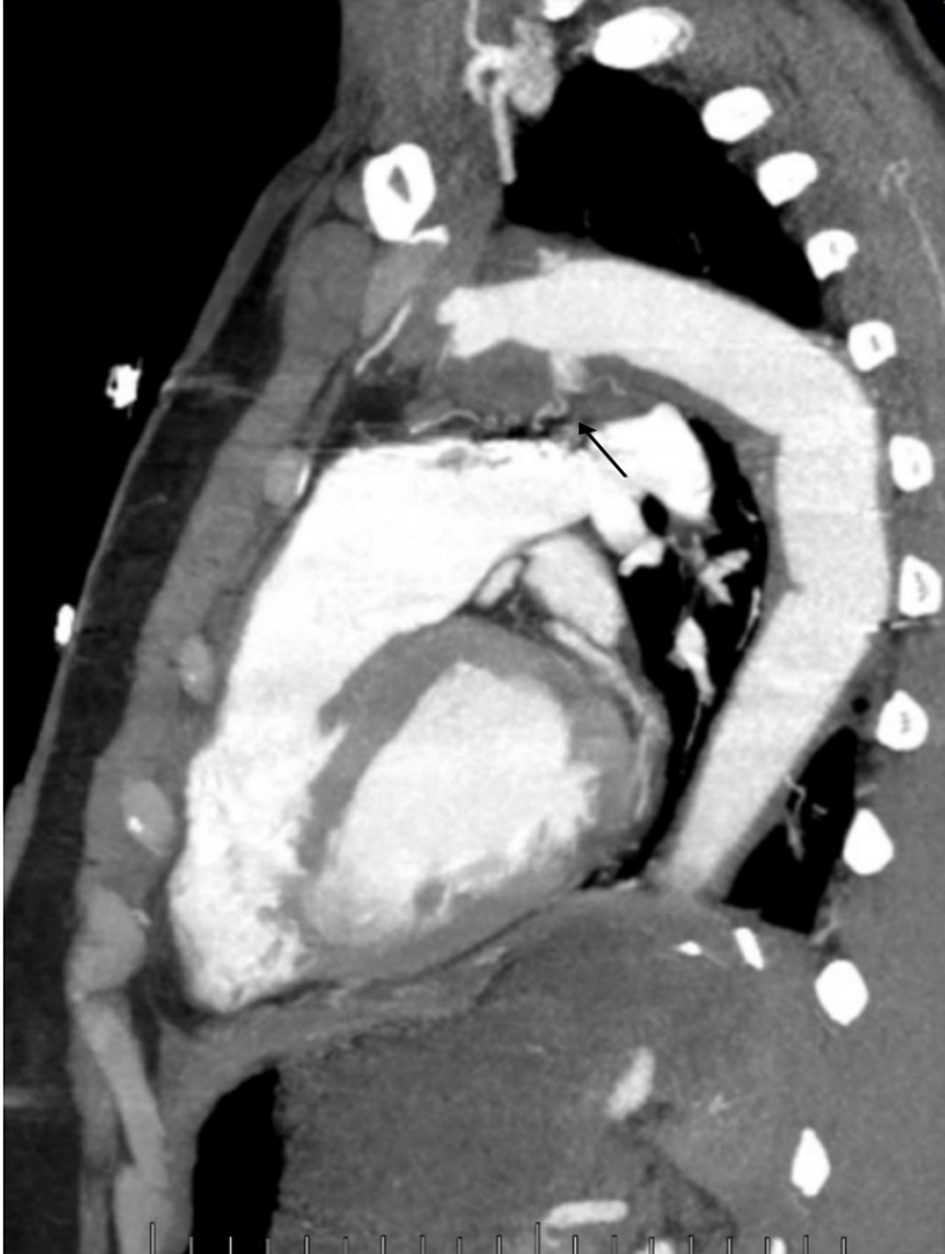
Contrast-enhanced CT of the chest ( sagittal view) The arrow shows the aortic arch with a peri-aortic thrombus.

He was treated for a non-ST-elevation myocardial infarction (NSTEMI), thrombolysis in myocardial infarction (TIMI) risk score of 2, with heparin for 48 hours. However, during this period, his troponins up-trended to 9.76, raising concern for an ongoing thromboembolic event. A CT angiogram of the chest was obtained, which was revealing for a partially thrombosed pseudoaneurysm along the aortic arch with dimensions of 5.2 x 4.9 cm, and, more significantly, a thrombus visualized adjacent to the contrast-filled aortic arch lumen with a combined diameter of 5.7 cm. Figures 1-3 demonstrate the aortic pseudoaneurysm and thrombus within the false lumen. 

His troponins subsequently trended downward with 48-hour anticoagulation. The patient underwent a repeat median sternotomy, evacuation of aortic pseudoaneurysm thrombus, and endovascular coiling with aortic repair via Dacron graft (GoreTex, Arizona, USA) anastomosis by cardiothoracic surgery. This patient’s post-operative course was uncomplicated, and he was followed closely outpatient by his cardiologist.

## Discussion

Our case illustrates some post-surgical complications of Marfan syndrome, which in this young patient presented as NSTEMI, aortic arch pseudoaneurysm, and aortic pseudoaneurysm thrombosis as complications after being lost to follow up with a cardiologist. 

Manifestations of Marfan syndrome include lesions affecting connective tissues, including ocular lens and osseous deformities, but virtually all adults with Marfan syndrome develop abnormalities of the cardiovascular system, which accounts for the vast majority of premature deaths in this cohort [5]. An autosomal dominant mutation initiates the pathogenetic sequence in the Fibrillin-1 gene. Its cardinal manifestations are comprised of proximal aortic aneurysm, dislocation of the ocular lens, and long-bone overgrowth. The cardiovascular lesions drive the prognosis in these patients. Even after aortic surgery, patients with Marfan syndrome remain at risk for new aortic pathology, including dissection and aneurysm, or propagation of a pre-existing aortic lesion [6]. The mean life expectancy of individuals with Marfan syndrome is 32 years if left untreated [7]. Therefore, life-long cardiovascular monitoring is warranted to prolong the survival of these patients. 

All of this patient’s complications secondary to Marfan syndrome, including acute myocardial infarction and pseudoaneurysm with thrombus formation, may have potentially been avoided, or at least mitigated, had he been monitored as per surveillance guidelines. Pseudoaneurysm formations in Marfan patients are life-threatening complications following thoracoabdominal repair. It has been estimated that 7%-25% of patients who undergo surgical replacement of the aortic valve, root, or ascending aorta experience pseudoaneurysm formation [6]. CT and magnetic resonance (MR) angiography (CTA and MRA, respectively) remain the mainstay for postoperative imaging modalities for surveillance of the aorta in Marfan syndrome and can demonstrate these complications [6]. Intermittent surveillance of the entire aorta with CT or MRA scans should be initiated in young adulthood for all individuals with Marfan syndrome, in addition to annual transthoracic echocardiograms [6]. Annual echocardiographic surveillance should be conducted to monitor the status of the ascending aorta when the aortic dimension is relatively small and the rate of aortic dilatation is relatively slow. More frequent evaluations by a cardiologist are indicated when the aortic root diameter exceeds 4.5 cm in adults, if the rate of aortic dilation exceeds 0.5 cm/year, or there is a severe or progressive valve/ventricular dysfunction [6]. This threshold is further reduced for pregnant patients with Marfan syndrome, who are at increased risk of aortic dissection given increased cardiac output and pressures sustained by the aorta [6]. 

## Conclusions

Mafran syndrome can lead to multiple reoccurring aortic diseases due to the abnormalities within the connective tissue. Our case emphasizes the importance of close follow-up with cardiology to lessen the consequences of the aforementioned difficulties and demonstrate the importance of a multidisciplinary approach to treating the intricacies of Marfan syndrome.

## References

[1] Keane MG, Pyeritz RE (2008). Medical management of Marfan syndrome. Circulation.

[2] (2020). Marfan syndrome. https://medlineplus.gov/genetics/condition/marfan-syndrome/.

[3] Dean JC (2002). Management of Marfan syndrome. Heart.

[4] Sulejmani F, Pokutta-Paskaleva A, Ziganshin B, Leshnower B, Iannucci G, Elefteriades J, Sun W (2017). Biomechanical properties of the thoracic aorta in Marfan patients. Ann Cardiothorac Surg.

[5] Judge DP, Dietz HC (2005). Marfan's syndrome. Lancet.

[6] Groner LK, Lau C, Devereux RB, Green DB (2018). Imaging of the postsurgical aorta in Marfan syndrome. Curr Treat Options Cardiovasc Med.

[7] von Kodolitsch Y, Raghunath M, Nienaber CA (1998). Marfan syndrome: prevalence and natural course of cardiovascular manifestations (Article in German). Z Kardiol.

